# Retraction: Effect of temperature & humdity on population dynamics of insects’ pest complex of cotton crop

**DOI:** 10.1371/journal.pone.0274235

**Published:** 2022-09-14

**Authors:** The PLOS ONE Editors

The *PLOS ONE* Editors retract this article [1] because it was identified as one of a series of submissions for which we have concerns about authorship, competing interests, and peer review. We regret that the issues were not addressed prior to the article’s publication.

At the time of retraction, the original article was republished to correct the affiliation for author Huma Khan.

MB responded but expressed neither agreement nor disagreement with the editorial decision. MAB, HK, MSN, HF, MH, SA, MAE-Z, RAA, MT and RJ did not reply or could not be reached.
